# Wear Behavior and Microstructure of Mg-Sn Alloy Processed by Equal Channel Angular Extrusion

**DOI:** 10.3390/ma10111315

**Published:** 2017-11-16

**Authors:** Jung-Hsuan Chen, Yen-Chen Shen, Chuen-Guang Chao, Tzeng-Feng Liu

**Affiliations:** 1 Department of Industrial Education, National Taiwan Normal University, Taipei 106, Taiwan; jhchen@ntnu.edu.tw; 2 Department of Materials Science and Engineering, National Chiao Tung University, Hsinchu 300, Taiwan; ycshen@nctu.edu.tw (Y.-C.S.); tfliu@nctu.edu.tw (T.-F.L.)

**Keywords:** Mg-Sn alloy, Equal Channel Angular Extrusion, pin-on-disc wear test, microstructure

## Abstract

Mg-5wt.% Sn alloy is often used in portable electronic devices and automobiles. In this study, mechanical properties of Mg-5wt.% Sn alloy processed by Equal Channel Angular Extrusion (ECAE) were characterized. More precisely, its hardness and wear behavior were measured using Vickers hardness test and a pin-on-disc wear test. The microstructures of ECAE-processed Mg-Sn alloys were investigated by scanning electron microscope and X-ray diffraction. ECAE process refined the grain sizes of the Mg-Sn alloy from 117.6 μm (as-cast) to 88.0 μm (one pass), 49.5 μm (two passes) and 24.4 μm (four passes), respectively. Meanwhile, the hardness of the alloy improved significantly. The maximum wear resistance achieved in the present work was around 73.77 m/mm^3^, which was obtained from the Mg-Sn alloy treated with a one-pass ECAE process with a grain size of 88.0 μm. The wear resistance improvement was caused by the grain size refinement and the precipitate of the second phase, Mg_2_Sn against the oxidation of the processed alloy. The as-cast Mg-Sn alloy with the larger grain size, i.e., 117.6 μm, underwent wear mechanisms, mainly adhesive wear and abrasive wear. In ECAE-processed Mg-Sn alloy, high internal energy occurred due to the high dislocation density and the stress field produced by the plastic deformation, which led to an increased oxidation rate of the processed alloy during sliding. Therefore, the oxidative wear and a three-body abrasive wear in which the oxide debris acted as the three-body abrasive components became the dominant factors in the wear behavior, and as a result, reduced the wear resistance in the multi-pass ECAE-processed alloy.

## 1. Introduction

Mg alloys are regarded as a material with great potential for high performance structural applications due to their low density, great mechanical properties, and good cast ability [1,2,3,4]. Mg-Sn alloys in particular have attracted much attention because the melting point of the intermetallic phase, Mg_2_Sn, in the Mg-Sn alloy (770 °C) is much higher than that of the Mg_17_Al_12_ phase in the Mg-Al alloy (462 °C) [5,6]. Therefore, the high temperature mechanical properties of Mg-Sn alloys can be improved substantially. Liu and his coworkers [7] demonstrated the microstructure, tensile properties, and creep behavior of as-cast Mg-(1–10%) Sn alloys. Their experiment results indicated that the Mg-55wt.% Sn alloy had the greatest tensile and elongation. So far, there has been much research on Mg-5wt.% Sn alloys focused on mechanical properties, high temperature performances, corrosion characteristics, creep properties, and so on [8,9,10,11]. Although many of studies have been conducted to date, research discussing the wear properties of Mg-5wt.% Sn is still limited. Wear control is a necessary consideration for the advanced and reliable machine. Wear rate, wear modes, and wear mechanism all should be considered when selecting suitable material for specific applications. In this study, we investigated the wear behavior of Mg-5wt.% Sn alloys, focusing on the alloys processed by Equal Channel Angular Extrusion (ECAE). ECAE is a technology used to refine grain size and therefore improve the mechanical properties of metals and alloys [12,13]. The influences of the ECAE process on the wear properties of Mg-Sn alloys and the wear mechanisms of these alloys during sliding were explored. Additionally, the relation between the microstructure of the alloys after ECAE and the mechanical properties of the alloys was examined.

## 2. Materials and Methods

Mg-5% Sn alloy was produced using the ingot casting method. Pure magnesium (99.95 wt.%) and pure tin (99.98 wt.%) were placed in a stainless steel crucible and melted in protective gas, SF_6_. The melt was superheated to 800 °C, and then at 720 °C, poured into a die steel mold with dimensions of 300 mm × 70 mm × 60 mm. For the ECAE process, as-cast Mg-Sn alloy specimens were machined into a billet with dimensions of 15 mm × 15 mm × 50 mm via wire electrical discharge machining, and then heated in the die for 20 min before the extrusion. The specimens were extruded in a sharp 120° angle ECAE die, which was preheated to 300 °C. The extrusion rate was 0.022 mm/s and the specimens were quenched in air after the ECAE process. Subsequent ECAE passes were performed at 200 °C and followed by a 90° rotation before the next extrusion process. ECAE at the lower temperature at later ECAE passes prevented the recrystallization of the Mg-Sn specimen and reduced the die frictional effects [14]. The specimens were processed for a total of one, two, and four passes using this method. The specimens for the wear test were machined into cylindrical pins (8 mm in diameter and 4 mm in height) via wire electrical discharge machining.

The microstructures of the as-cast and ECAE-processed Mg-Sn alloy specimens were investigated by scanning electron microscope (SEM, JEOL, Tokyo, Japan) and X-ray diffraction (XRD, Bruker, Billerica, MA, USA). The hardness and wear behavior were measured using Vickers hardness test and the dry sliding wear test. The wear test was operated without any lubricants using a conventional pin-on-disc testing machine (Plint, Berkshire, UK) at ambient temperature. The testing specimen, Mg-Sn alloy, was used as the pin and a SKD11 tool steel with hardness about HRC (Rockwell C Hardness) 60 was used as the disc-shaped counter material. The track radius was 10 mm and the sliding velocity was maintained at 1.2 m/s. The load was 29.4 N over a distance of 2500 m. Surfaces of the worn specimens and the wear debris were observed by SEM and XRD to clarify the wear mechanisms.

## 3. Results and Discussion

**Figure 1:**
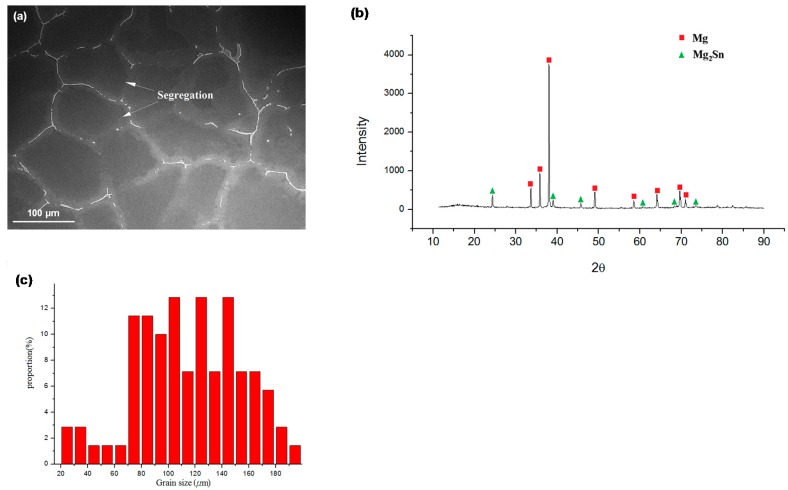
(**a**) Microstructure of the as-cast Mg-Sn alloy; (**b**) XRD spectrum of the as-cast Mg-Sn alloy; (**c**) grain size distribution of the as-cast Mg-Sn alloy calculated from the microstructure image.

**Figure 2:**
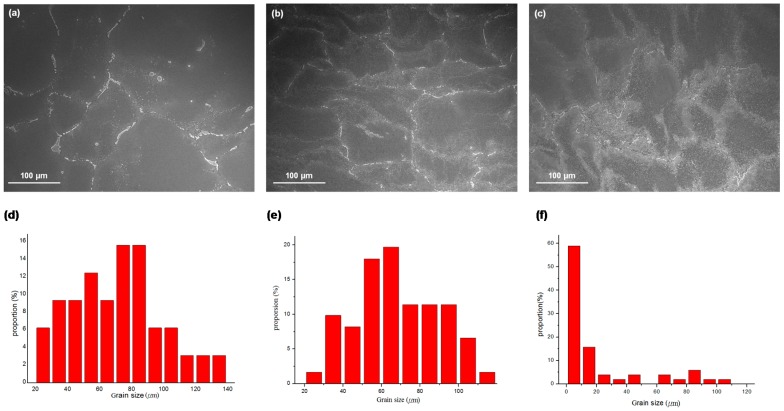
Microstructures of Equal Channel Angular Extrusion (ECAE)-processed Mg-Sn alloys: (**a**) one-pass (1N); (**b**) two-pass (2N); and (**c**) four-pass (4N), and grain size distribution of ECAE-processed Mg-Sn alloys calculated from the microstructure images: (**d**) one-pass (1N); (**e**) two-pass (2N); and (**f**) four-pass (4N).

The microstructure of the as-cast Mg-Sn alloy was observed via SEM. Figure 1a shows continuous eutectic structures at the grain boundary and a higher Sn content than that of the matrix in the chemical compositions of the precipitates. There were obvious segregations in the as-cast Mg-Sn alloy. According to the XRD analysis shown in Figure 1b, the precipitate was the intermetallic compound Mg_2_Sn, and the matrix was α-Mg. Further image analysis characterized the mean grain size and the size distribution of the Mg-Sn alloy. Histogram analysis showed the corresponding grain size distribution of the as-cast Mg-Sn alloy, as presented in Figure 1c. Abroad grain size distribution was observed from 20 μm to 200 μm, and the average grain size was around 117.6 μm. Microstructures of ECAE-processed specimens were also observed using SEM in order to evaluate the changes in grain size, the precipitate concentration, and the uniformity of the microstructures. Figure 2 shows that a more homogeneous microstructure, a finer grain size, and more uniform grain size distribution was achieved with a higher number of ECAE passes. The eutectic structures composed of α-Mg and Mg_2_Sn were shattered and dispersed uniformly in the grain and grain boundary after ECAE processing. The average grain sizes of one-, two-, and four-pass ECAE-processed alloys in Figure 2d–f were 88.0 μm, 49.5 μm, and 24.4 μm, respectively.

**Figure 3:**
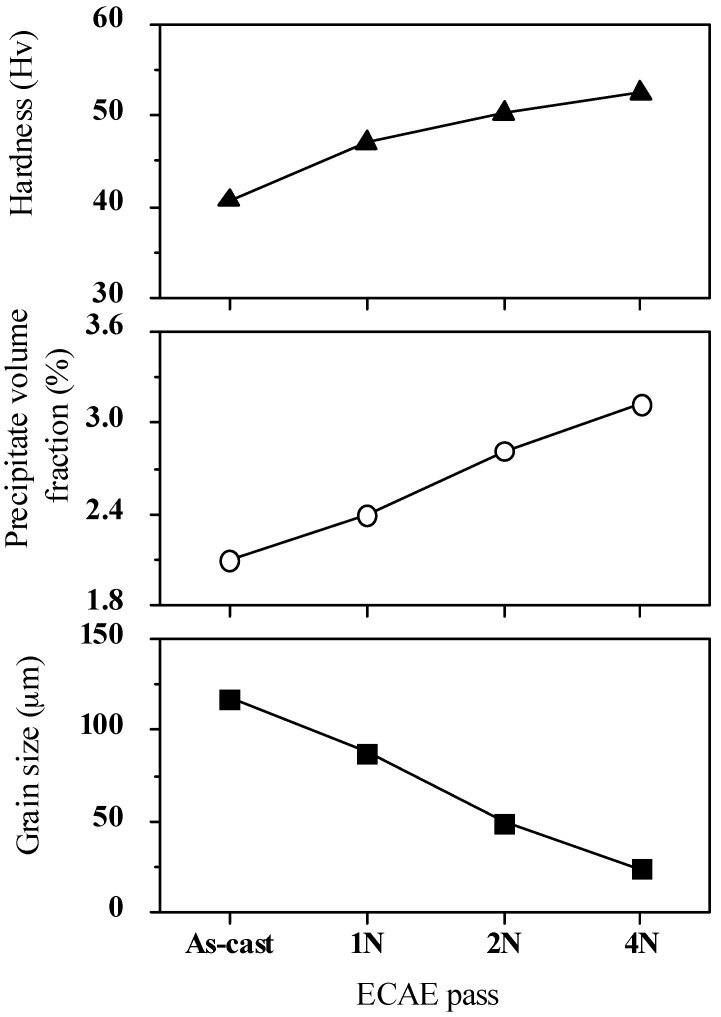
Grain size, hardness, and precipitate fraction of the Mg-Sn alloys before and after ECAE processing.

The relation among the grain size, the precipitate fraction of Mg_2_Sn, and the hardness of the Mg-Sn alloys after ECAE processing were investigated in Figure 3. The highest hardness, 52.5 Hv, could be obtained in the Mg-Sn alloy with four-pass ECAE processing, for which average grain size was 24.4 μm and the precipitate fraction was 3.12%. The enhancement of the hardness was attributed to the grain refinement and the precipitation of the second phase, Mg_2_Sn, after ECAE processing. A larger amount of grain boundaries and precipitates would disturb the movements of the dislocations, leading to the increase of the hardness.

The wear behaviors of ECAE-processed Mg-Sn alloys were investigated by pin-on-disc experiments. The effect of the grain size on the mechanical properties of a material can be evaluated by the Hell-Petch relationship [15,16,17]:

(1) $$H=H_{0}+k/\sqrt{d},$$

*H* is the measured hardness of the material, *H*_0_ is the intrinsic hardness dependent on frictional lattice resistance to dislocation motion, *d* is the average grain size, and *k* is the material-specific strengthening coefficient. According to the equation, the material with finer grains has better mechanical properties such as strength and hardness compared to that with coarse grains. This is in agreement with our aforementioned findings. The relation between the wear resistance and the hardness can be calculated by the Archard equation [18,19]:

(2) $$W_{\mathrm{Resistance}}=K\frac{H}{N}$$

where *K* is the wear coefficient, *H* is the hardness, and *N* is the applied load during sliding. At a constant applied load, the wear resistance would enhance when the hardness of the material increases.

**Figure 4:**
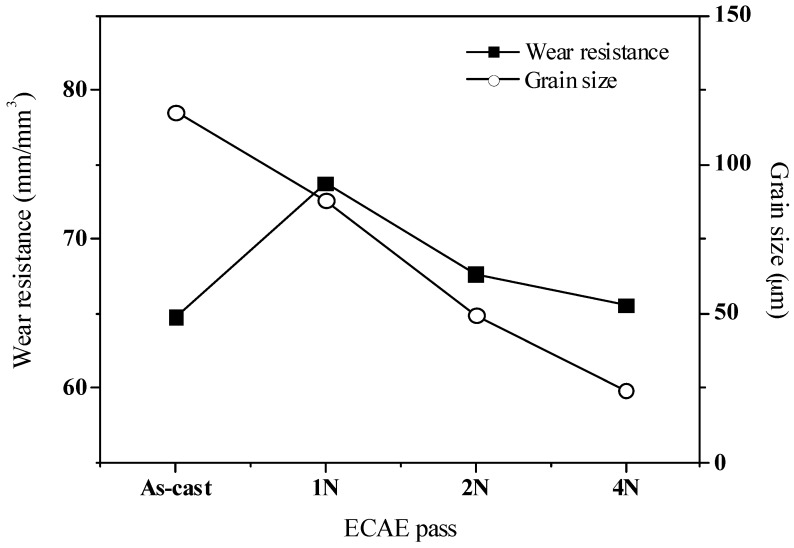
The wear resistance of the as-cast and ECAE-processed Mg-Sn alloys.

Figure 4 shows the wear resistances of the as-cast and ECAE-processed Mg-Sn alloys. The maximum value of the wear resistance was 73.77 m/mm^3^ obtained from one-pass ECAE-processed alloy, for which the grain size was 88.0 μm and the precipitate fraction was 2.4%. As the number of ECAE process passes increased, the grain refined and the precipitate increased; however, the wear resistance worsened. Since the wear resistance did not improve as grain size became smaller, the wear performance was not only affected by the mechanical properties including the strength and hardness, which were controlled by the grain refinement and the precipitate fraction. Similar wear behaviors were observed in Ti and the eutectic Al-Si alloy [14,20]. Kucukomeroglu [20] found that the ECAE process decreased the wear resistance of the Al-Si alloy in the oxidative wear during sliding, although there was improvement in strength and ductility values. He also pointed out that the oxidation rate in the ECAE-processed alloy was higher than that in the as-cast alloy as a result of the high internal energy due to the high dislocation density and the production of a stress field during the severe plastic deformation by multi-pass ECAE. Therefore, the oxygen layer on the surface of the alloy may play a crucial role in the wear performance. As for the Mg-Sn alloy, Yu and his coworkers [21] observed that the surface oxide film mainly composed of MgO was relatively thin and loose. Therefore, the high temperature oxide film of the Mg-Sn alloy could not protect its matrix. The oxide film would be disrupted and easily removed by spalling or fragmentizing during sliding under high normal or shear stress. This phenomenon would result in oxidative wear and a three-body abrasive wear, where oxide debris acted as three-body abrasive components [1,14,21]. The energy-dispersive X-ray spectroscopy (EDS) analysis of the worn surfaces of the as-cast and four-pass ECAE-processed alloys were conducted in this experiment, as shown in Table 1. The four-pass ECAE-processed alloy had a higher oxygen signal than the as-cast alloy. This indicated that a thicker oxide film formed on the surface of the EACE-processed alloy during sliding. The iron signals on the surface came from the counter material by the abrasion during sliding.

We have shown that grain size refinement, the precipitate of the harder second phase, and the effect of oxidation on the surface seem to be the dominant factors affecting the wear resistance of ECAE-processed alloys. Combining the contributions of these three factors could explain why the maximum wear resistance was obtained at the alloy undergoing the one-pass ECAE process in this study.

**Figure 5:**
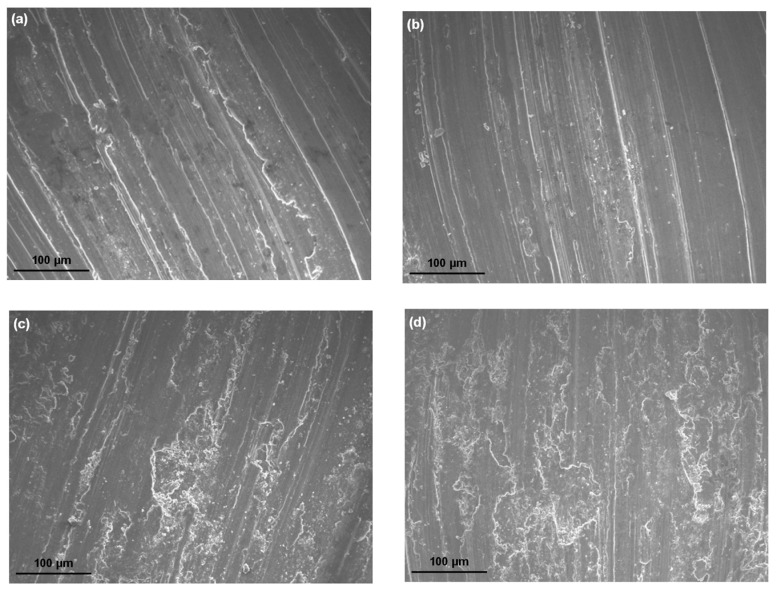
Worn surface morphologies of the Mg-Sn alloys after the pin-on-disc wear test: (**a**) as cast; (**b**) one-pass (1N); (**c**) two-pass (2N); and (**d**) four-pass (4N).

**Figure 6:**
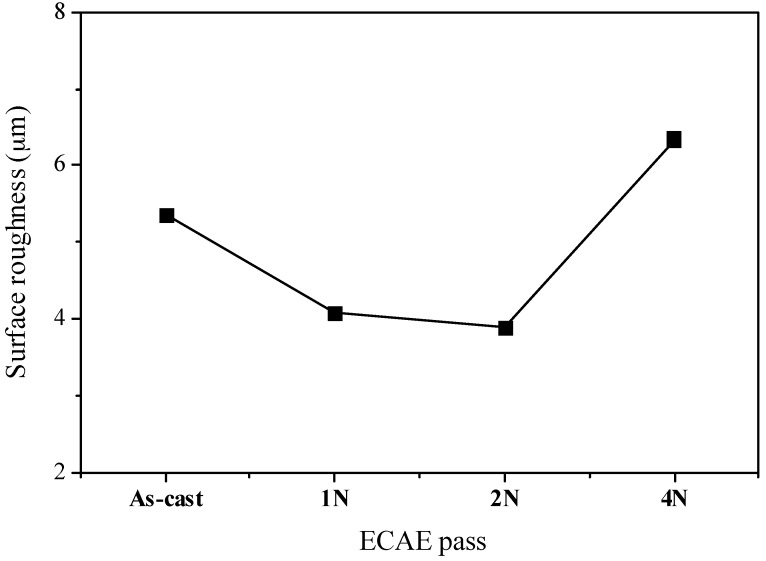
The surface roughness of the as-cast and ECAE-processed Mg-Sn alloys after the wear test.

**Figure 7:**
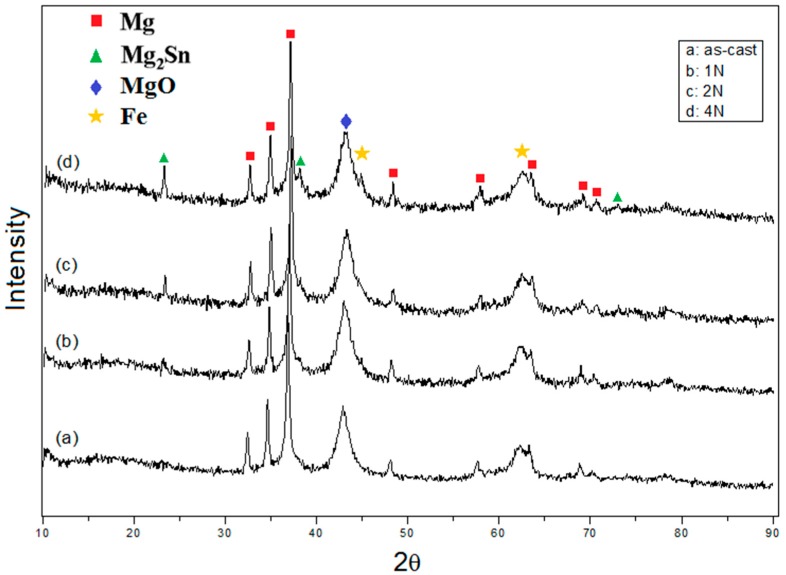
XRD analyses of the wear debris: (**a**) as-cast; (**b**) one-pass (1N); (**c**) two-pass (2N) and (**d**) four-pass (4N).

In order to investigate the wear mechanism, the surfaces of the alloys after sliding were examined by SEM. The worn surfaces of the Mg-Sn alloys showed diverse topographical features, as seen in Figure 5. Figure 5a shows the obvious scratches due to the abrasive wear on the worn surface of the as-cast alloy. Moreover, the marks of adhesive wear with the plastic deformation were observed near the region of the ploughing. As the grain size reduced to 88μm, leading to the increase of the strength, the worn surface of the one-pass ECAE-processed alloy in Figure 5b showed abrasive damages such as the microcutting and ploughing of the solid without the plastic deformation. Therefore, the main wear mechanism was the abrasive wear in the alloy with one-pass ECAE. When the grain size continued to refine, the influence of the surface oxidation appeared gradually. The surface oxidation rate increased with the increase of the ECAE pass number and a three-body abrasive wear tended to take place during sliding. Meanwhile, more precipitates produced and acted as asperities that were presumed to fracture easily. The abrasive components in the form of particles or thin flakes were trapped between two surfaces (pin and disc) in relative motion. The generation of fatigue cracks after repeated stress was attributed to the surface traction and the plastic deformation. The cracks propagated parallel to the surface, delaminated, and formed loose debris. Therefore, there were many cavities and craters with irregular shapes on the worn surface of the four-pass ECAE-processed alloy, as shown in Figure 5d. Figure 6 shows the surface roughness of the worn surfaces of the as-cast and ECAE-processed alloys. The as-cast alloy with the grain size of 117.6 μm easily exhibited deep grooves during sliding due to the soft matrix. The roughness of the as-cast alloy was measured as 5.47 μm. After ECAE processing, the grain size reduced, the precipitate increased, and the surface oxidation occurred gradually during sliding. The main wear mechanisms became the abrasive wear and the oxidative wear. Therefore, the worn surface was smoother than the as-cast alloy and the surface roughness decreased to around 4 μm. On the four-pass ECAE-processed alloy, delaminations and fractures due to the severe fatigue and oxidative wear were observed. As a result, the surface roughness was increased to 6.5 μm. Wear debris produced during the wear test were collected and examined using XRD. Figure 7 presents the XRD spectra from the wear debris of the as-cast and ECAE-processed alloys. The debris had a significant signal of MgO oxide, suggesting that evident oxidation had occurred during sliding. Moreover, the iron peak from the counter material by the abrasion was found in the four-pass ECAE-processed alloy due to the increase of the precipitate fraction and the appearances of a large number of MgO oxides.

## 4. Conclusions

In this study, Mg-5% Sn alloy was fabricated using the ingot castingtechnique, and then extruded by the ECAE process. Differences in microstructure and material properties between the as-cast alloy and ECAE-processed alloys were investigated. The experimental results showed that the precipitates, Mg_2_Sn, increased and distributed uniformly due to the shear stress produced during the ECAE process. Meanwhile, the grain size refined significantly. The highest hardness, 52.5 Hv, could be obtained in the Mg-Sn alloy treated withthe four-pass ECAE process, forwhich average grain size was 24.4 μm and the precipitate fraction was 3.12%. In this study, Mg-Sn alloy subjected to the one-pass ECAE process had the maximum wear resistance of 73.77 m/mm^3^, a grain size of 88.0 μm, and the precipitate fraction of 2.4%. As the number of ECAE passes increased, so did the internal stress. This could be explained by the high dislocation density and stress field in the ECAE process, which caused the Mg-Sn alloy to oxidize quickly during the wear test. The main wear mechanisms changed fromadhesion and abrasion to oxidative wear and three-body abrasive wear, in which the oxide debris acted as the three-body abrasive components, leading to delaminations and fatigue fractures. Therefore, the wear performance reduced significantly in the multi-pass ECAE-processed alloy.

## Figures and Tables

**Table 1:** EDS results obtained from the as-cast and four-pass ECAE-processed alloys.

As-Cast Alloy		ECAE-Processed Alloy (4N)	
Element	Weight %	Element	Weight %
C	6.60	C	4.99
O	15.00	O	32.34
Mg	75.23	Mg	57.33
Sn	3.17	Sn	3.09
-	-	Fe	2.24
